# Epigenetic prediction of complex traits and death

**DOI:** 10.1186/s13059-018-1514-1

**Published:** 2018-09-27

**Authors:** Daniel L. McCartney, Robert F. Hillary, Anna J. Stevenson, Stuart J. Ritchie, Rosie M. Walker, Qian Zhang, Stewart W. Morris, Mairead L. Bermingham, Archie Campbell, Alison D. Murray, Heather C. Whalley, Catharine R. Gale, David J. Porteous, Chris S. Haley, Allan F. McRae, Naomi R. Wray, Peter M. Visscher, Andrew M. McIntosh, Kathryn L. Evans, Ian J. Deary, Riccardo E. Marioni

**Affiliations:** 1 Centre for Genomic and Experimental Medicine, Institute of Genetics and Molecular Medicine, University of Edinburgh, Edinburgh, EH4 2XU UK; 2 Centre for Cognitive Ageing and Cognitive Epidemiology, University of Edinburgh, Edinburgh, EH8 9JZ UK; 3 Department of Psychology, University of Edinburgh, Edinburgh, EH8 9JZ UK; 4 Institute for Molecular Bioscience, University of Queensland, Brisbane, QLD Australia; 5 Aberdeen Biomedical Imaging Centre, Lilian Sutton Building, University of Aberdeen, Foresterhill, Aberdeen, AB25 2ZD UK; 6 Division of Psychiatry, University of Edinburgh, Royal Edinburgh Hospital, Edinburgh, EH10 5HF UK; 7 MRC Human Genetics Unit, Institute of Genetics and Molecular Medicine, University of Edinburgh, Edinburgh, EH4 2XU UK

**Keywords:** DNA methylation, Polygenic scores, Prediction, Ageing, Mortality

## Abstract

**Background:**

Genome-wide DNA methylation (DNAm) profiling has allowed for the development of molecular predictors for a multitude of traits and diseases. Such predictors may be more accurate than the self-reported phenotypes and could have clinical applications.

**Results:**

Here, penalized regression models are used to develop DNAm predictors for ten modifiable health and lifestyle factors in a cohort of 5087 individuals. Using an independent test cohort comprising 895 individuals, the proportion of phenotypic variance explained in each trait is examined for DNAm-based and genetic predictors. Receiver operator characteristic curves are generated to investigate the predictive performance of DNAm-based predictors, using dichotomized phenotypes. The relationship between DNAm scores and all-cause mortality (*n* = 212 events) is assessed via Cox proportional hazards models. DNAm predictors for smoking, alcohol, education, and waist-to-hip ratio are shown to predict mortality in multivariate models. The predictors show moderate discrimination of obesity, alcohol consumption, and HDL cholesterol. There is excellent discrimination of current smoking status, poorer discrimination of college-educated individuals and those with high total cholesterol, LDL with remnant cholesterol, and total:HDL cholesterol ratios.

**Conclusions:**

DNAm predictors correlate with lifestyle factors that are associated with health and mortality. They may supplement DNAm-based predictors of age to identify the lifestyle profiles of individuals and predict disease risk.

**Electronic supplementary material:**

The online version of this article (10.1186/s13059-018-1514-1) contains supplementary material, which is available to authorized users.

## Background

DNA-based predictors of health and lifestyle have potential uses in both clinical and non-clinical contexts. For example, biological predictors of smoking status and alcohol consumption may provide more accurate measurements than self-report, thereby improving disease prediction and risk stratification [1]. Here, using whole blood-derived samples, we develop ten novel DNA methylation-based predictors of modifiable health and lifestyle factors including alcohol consumption, smoking status, body mass index (BMI), waist-to-hip ratio, four measures of cholesterol, percentage body fat, and educational attainment. We then relate these predictors to both a health outcome (mortality) and lifestyle characteristics in an independent cohort.

DNA methylation (DNAm) is a commonly studied epigenetic modification characterized by chemical changes to DNA, typically at a cytosine-phosphate-guanine (CpG) nucleotide base pairing [2]. These modifications are dynamic, tissue-specific, and cell-specific [3], are involved in gene regulation, and can be influenced by both genes and the environment [4].

Through large meta-analysis projects, methylation signals at individual CpG sites have been associated with educational attainment, smoking, alcohol consumption, cholesterol levels, and BMI [5–13]. Such studies have also used methylation predictors (from a combination of CpG sites) to predict the phenotype of interest in independent cohorts. For example, 7% of the variance in BMI and 2% of the variance in educational attainment can be explained by their respective predictors [5, 14]. Moreover, DNA methylation has been reported to explain 0.74% and 9.51% of the variation in total and high-density lipoprotein (HDL) cholesterol levels, respectively [11]. Studies have also combined genetic risk scores into their prediction models, showing that the DNAm predictors contribute independently to the variance explained in BMI and C-reactive protein levels [14, 15]. Moreover, single CpG sites and DNAm predictors of smoking have been linked to lung cancer/mortality [16], while DNAm-based predictors of BMI and inflammation have been linked to cardiometabolic traits [7, 15].

There are, however, several limitations to existing studies. First, the CpG weights for the predictors are derived separately for each CpG, which does not account for their inter-correlations. Second, large samples are required to generate precise weights. This has meant conducting meta-analyses with data from heterogeneous populations where different quality control metrics have been applied. Third, the CpG prediction weights are typically based on Z-scores rather than effect sizes, that is, the trait was modelled as the predictor with the CpG as the outcome in the epigenome-wide association studies (EWASs). These Z-score weights are equivalent to modelling by *p* values, which do not account for the magnitude of the CpG-trait association. Fourth, arbitrary significance threshold cut-offs are used to select the number of CpGs used in each predictor rather than training a predictor on an optimized set of CpGs.

Here, we overcome the above limitations as described below. We model all CpGs simultaneously in a single large cohort of over 5000 individuals. We model the traits of interest as the outcomes and the CpGs as the predictors and train optimized predictors using penalized regression methods. We then apply these predictors to an independent cohort study of approximately 900 individuals to determine: (1) the proportion of variance the DNAm predictors explain in the outcomes; (2) the extent to which these proportions are independent from the contribution of genetics; (3) the accuracy with which the DNAm predictors can identify obese individuals, college-educated individuals, heavy drinkers, high cholesterol levels, and current smokers if provided with a random DNA sample from the population; and (4) the extent to which they can predict health outcomes, such as mortality, and if they do so independently from the phenotypic measure.

## Results

Summary information on the ten phenotypes in both the training (Generation Scotland: The Scottish Family Health Study [GS]) and test (The Lothian Birth Cohort 1936 [LBC1936]) datasets is presented in Table 1. LBC1936 is an older cohort than GS (mean age 70 vs 49 years), with a more even gender balance (51% vs 39% male). LBC1936, when compared with GS participants, had around two fewer years of education, were of similar mean BMI (both cohort means were ~ 27 kg/m^2^), drank slightly less alcohol (median difference of 3 units per week), had a lower ratio of current to never smokers (20% vs 27%), lower levels of low-density lipoprotein (LDL) (with remnant) cholesterol (mean difference of 0.3 mmol/L), higher total cholesterol (mean difference of 0.3 mmol/L) a higher ratio of total:HDL cholesterol (mean difference of 0.1), and similar levels of HDL cholesterol (mean level of 1.5 mmol/L).

**Table 1:** Summary of the Generation Scotland (GS) and Lothian Birth Cohort 1936 (LBC1936) studies

	GS			LBC 1936		
	*N*	Mean	SD	*N*	Mean	SD
Age (years)	5087	48.5	14.0	895	69.6	0.8
Body mass index (kg/m^2^)	5036	27.0	5.2	894	27.8	4.4
Total cholesterol (mmol/L)	4200	5.1	1.1	885	5.4	1.2
HDL cholesterol (mmol/L)	4192	1.5	0.4	812	1.5	0.4
LDL with remnant cholesterol (mmol/L)	4192	3.6	1.1	812	3.9	1.1
Total:HDL cholesterol ratio (ratio)	4192	3.7	1.2	809	3.8	1.1
Waist-to-hip ratio (ratio)	4984	0.9	0.1	–	–	–
Body fat (%)	4950	30.8	9.6	–	–	–
	N	Median	Q1, Q3	N	Median	Q1, Q3
Alcohol (units per week)	2819	8	2, 15	895	5	0.5, 14
Education (years)^*^	4804	12–13	10–11, 16–17	895	10	10, 12
	N	%		N	%	
Sex						
Male	1956	38.5		453	50.6	
Female	3131	61.5		442	49.4	
Smoking						
Never smoked	2523	73.3		418	46.7	
Ex-smoker	–	–		375	41.9	
Current smoker	921	26.7		102	11.4	

Sample counts are provided for age, sex, and measures for health and lifestyle factors in both the GS and LBC1936 studies

*Education was measured as an ordinal variable: 0, 0 years; 1, 1–4 years; 2, 5–9 years; 3, 10–11 years; 4, 12–13 years; 5, 14–15 years; 6, 16–17 years; 7, 18–19 years; 8, 20–21 years; 9, 22–23 years; 10, ≥ 24 years

The LASSO regressions returned predictors based on 204–1109 CpGs. The regression weights for the predictors are shown in Additional file 1: Tables S1–S10. DNAm predictors for the ten variables were created in LBC1936 at the baseline wave, at a mean age of approximately 70 years (*n* = 895).

Correlations between the phenotypic measures in GS are presented in Additional file 2: Figure S1. Correlations between the phenotypic measures, genetic measures, and DNAm predictors in LBC1936 are presented in Additional file 2: Figures S2–S4. Strong correlations were seen between the DNAm scores for cholesterol variables (*r* = − 0.6–0.8) and BMI and body fat percentage (*r* = 0.9). There was a negative correlation between DNAm scores for smoking and education (*r* = − 0.5). The phenotypic smoking:DNAm education association was of a similar magnitude (*r* = − 0.4) Correlations between polygenic scores were generally weak, with the exception of scores for LDL with remnant cholesterol and total cholesterol (*r* = 0.8), and BMI and body fat percentage (*r* = 0.4).

### DNAm predictors explain phenotypic variation

**Fig. 1:**
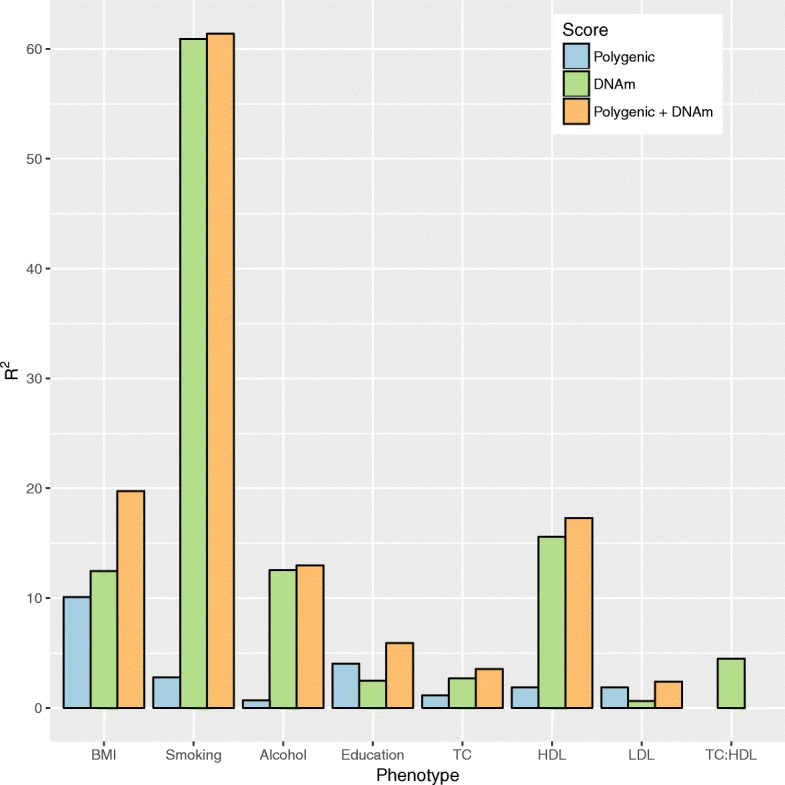
DNAm and polygenic prediction of health and lifestyle factors. Proportion of phenotypic variance explained (R^2^) is plotted for eight traits: BMI; smoking; alcohol consumption (alcohol); education; total cholesterol (TC); HDL cholesterol (HDL); LDL with remnant cholesterol (LDL); and total:HDL cholesterol ratio (TC:HDL) based on each trait’s polygenic score (*blue*), DNA methylation-based score (*green*), and additive genetic + epigenetic score (*orange*)

Age and sex-adjusted linear regression models showed that the DNAm predictors, which were developed in GS, explained a small proportion of the phenotypic variance in educational attainment, total cholesterol, cholesterol ratios, and LDL with remnant cholesterol (0.6–4.5%); a moderate proportion of the variance in BMI, HDL cholesterol, and alcohol consumption (12.5–15.6%); and a high proportion of the variance in smoking (60.9%; Table 2; Fig. 1).

**Table 2:** Predicting LBC1936 phenotypes using methylation and genetic predictors for health and lifestyle factors

Trait	DNAm score (%)	Polygenic score (%)	DNAm + polygenic (%)
BMI (kg/m^2^)	12.5	10.1	19.7
Alcohol (units per week)	12.5	0.7	13.0
Smoking (current/ever/never)	60.9	2.8	61.4
Educational attainment (years)	2.5	4.0	5.9
Total cholesterol (mmol/L)	2.7	1.1	3.6
HDL cholesterol (mmol/L)	15.6	1.9	17.3
LDL with remnant cholesterol (mmol/L)	0.6	1.8	2.4
Total:HDL cholesterol ratio	4.5	–	–
Waist-to-hip ratio	–	–	–
Body fat (%)	–	–	–

For each trait, the proportion of phenotypic variance explained is presented for DNAm score, polygenic score, and combined DNAm + polygenic scores for health and lifestyle factors

The corresponding polygenic scores explained a small proportion of the phenotypic variance in alcohol consumption, education, smoking, and total cholesterol, HDL cholesterol, and LDL with remnant cholesterol (0.7–4.0%). A moderate proportion of the phenotypic variance in BMI was explained by the BMI polygenic score (10.1%; Table 2; Fig. 1). Models including both the DNAm predictor and the polygenic score explained the most variance in each trait (Table 2; Fig. 1).

Phenotypes for body fat percentage and waist-to-hip ratio were not available in LBC1936. It was therefore not possible to assess the proportion of phenotypic variance explained by their DNAm and polygenic scores. Moreover, due to the absence of GWAS data for total:HDL cholesterol ratios, it was only possible to assess the proportion of variance explained by its DNAm score.

### DNAm predictors classify phenotype extremes

**Fig. 2:**
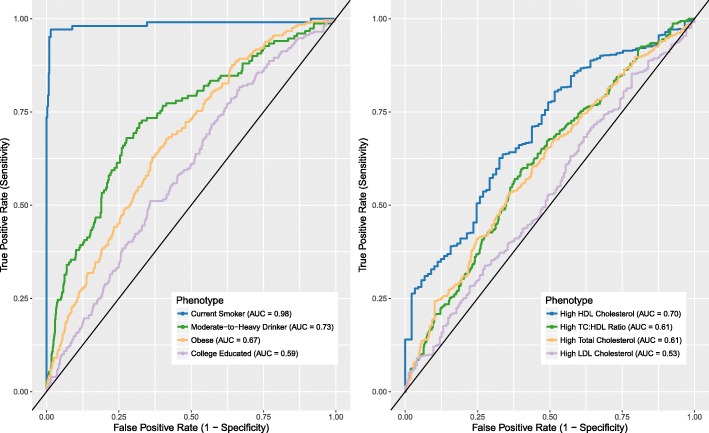
ROC analysis for DNAm predictors of alcohol, smoking, education, BMI, and cholesterol-related variables. Shown are ROC curves for predicting alcohol consumption, smoking status, obesity, and education (*left*), and cholesterol levels (*right*) Obese and non-obese are defined as BMI > 30 and ≤ 30 kg/m^2^; moderate-to-heavy and non-to-light drinkers defined as drinking > 21 and ≤ 21 units (men) or > 14 and ≤ 14 units (women) of alcohol per week; highly educated individuals had > 11 years of full-time education, compared to low-to-average education (≤ 11 years). High cholesterol levels were defined based on NHS guidelines (https://www.nhs.uk/conditions/high-cholesterol/: > 5 mmol/L for total cholesterol, > 3 mmol/L for LDL cholesterol, > 1 mmol/L for HDL cholesterol, and ≥ 4 for total:HDL cholesterol ratios)

For the area under the curve (AUC) analyses that predicted the binary classified phenotypes in LBC1936, there were 652 controls and 242 cases for obesity, 745 light-to-moderate drinkers and 150 heavy drinkers, 418 non-smokers and 102 current smokers, and 229 and 666 individuals with > 11 and ≤ 11 years of full-time education, respectively. Following dichotomization of the cholesterol-related variables, there were 531 and 354 individuals with high and low total cholesterol, respectively; 89 and 723 individuals with high and low HDL cholesterol, respectively; 637 and 175 individuals with high and low LDL with remnant cholesterol, respectively; and 307 and 502 with high and low total:HDL cholesterol ratios, respectively. There was near-perfect discriminatory power for the identification of current smokers (AUC = 0.98; 95% confidence interval [CI] = 0.97–1.00, Fig. 2) and moderate discrimination of obesity from non-obesity (AUC = 0.67; 95% CI = 0.63–0.71), high HDL levels from low HDL levels (AUC = 0.70, 95% CI = 0.64–0.75,) and of light-to-moderate drinkers from heavy drinkers (AUC = 0.73; 95% CI = 0.69–0.78). There was poor discrimination of those with more years of full-time education (AUC = 0.59; 95% CI = 0.55–0.63, Fig. 2), and higher total cholesterol, LDL with remnant cholesterol and total:HDL cholesterol ratios (total cholesterol AUC = 0.61; 95% CI = 0.57–0.64; LDL with remnant cholesterol AUC = 0.53; 95% CI = 0.48–0.58; total:HDL cholesterol ratio AUC = 0.61, 95% CI = 0.57–0.65). Including the polygenic scores in addition to the DNAm predictors improved the prediction of all traits, with the exception of alcohol consumption and total cholesterol (Additional file 3: Table S11). The smoking DNAm predictor was a significant addition to a logistic regression model for the binary education measure (smoking DNAm *p* = 0.006, education DNAm *p* = 0.08, and polygenic education *p* = 1.4 × 10^−8^) and high/low total cholesterol (smoking DNAm *p* = 0.033, total cholesterol DNAm *p* = 1.0 × 10^−6^, polygenic total cholesterol *p* = 0.014).

### DNAm predictors and mortality

**Fig. 3:**
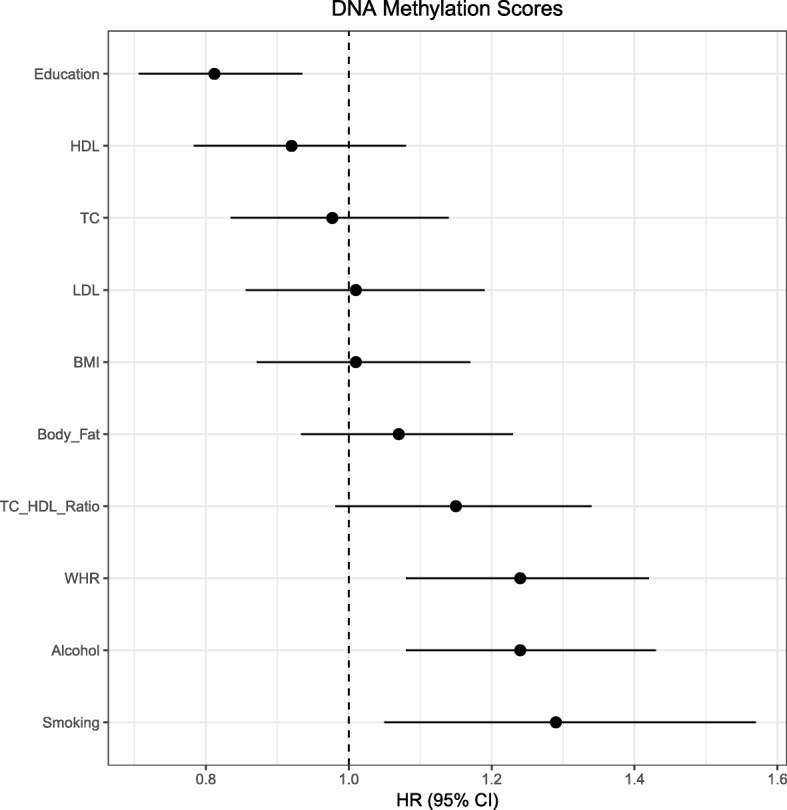
HRs for epigenetic (DNAm) predictors of mortality. *Forest plots* show HRs for DNAm scores for health and lifestyle factors. Effect sizes are per standard deviation with the exception of phenotypic smoking, for which never smokers are used as a reference group. *Horizontal lines* represent 95% CIs

Mortality in LBC1936 was assessed in relation to phenotype, DNAm scores, and polygenic scores using Cox proportional-hazards models, adjusting for age, sex, white blood cell proportions and each trait’s corresponding phenotype and polygenic score, where applicable (Additional file 3: Table S12 and Fig. 3). There were 212 deaths from 895 participants over 12 years of follow-up.

Higher phenotypic former smoking status (compared to never smokers) were associated with higher mortality risk (hazard ratio [HR] 1.45, 95% CI = 1.01–2.07, *p* = 0.044). A mild protective effect was associated with higher total cholesterol (HR = 0.86, 95% CI = 0.74–1.00, *p* = 0.047). No significant associations were observed in LBC1936 between risk of mortality and phenotypic BMI, alcohol consumption, educational attainment, or the remaining cholesterol-related variables. A significant association was observed between mortality and the polygenic score for body fat percentage (HR = 1.18, 95% CI = 1.03–1.36, *p* = 0.016) but not for the other eight genetic scores. Higher mortality risk was associated with higher DNAm scores for smoking (HR = 1.29, 95% CI = 1.05–1.57, *p* = 0.013), waist-to-hip ratio (HR = 1.24, 95% CI = 1.08–1.42, *p* = 0.002), and alcohol consumption (HR = 1.24, 95% CI = 1.08–1.43, *p* = 0.003). A higher DNAm score for education was associated with lower mortality risk (HR = 0.81. 95% CI = 0.71–0.93, *p* = 0.004). Following correction for multiple testing, DNAm signatures for education, alcohol consumption, and waist-to-hip ratio remained significantly associated with mortality (*p* < 0.05/10 = 0.005).

A final set of nine survival models were considered. These covaried for the smoking DNAm predictor alongside the covariates listed above (Table 3). Both the phenotypic BMI and smoking DNAm predictor were significant predictors of mortality (BMI HR = 1.23, 95% CI = 1.06–1.42, *p* = 0.005; DNAm smoking HR = 1.57, 95% CI = 1.39–1.78, *p* = 8.3 × 10^−13^). The association between the waist-to-hip ratio DNAm predictor and mortality remained after conditioning on the smoking DNAm predictor (*p* = 0.012). However, conditioning on the smoking DNAm predictor attenuated the association between the both the alcohol consumption and education DNAm predictors and mortality (alcohol consumption *p* = 0.134, education *p* = 0.352). Forest plots for phenotypic and genetic scores are available in Additional file 2: Figures S5 and S6.

**Table 3:** Cox proportional hazards survival models output for phenotypic, epigenetic (DNAm), and genetic (polygenic) predictors of health and lifestyle factors, conditioned on the smoking DNAm score

Trait	Predictor	HR	95% CI	*P*
Alcohol	Phenotypic	0.95	0.83–1.09	0.457
	Epigenetic	1.11	0.97–1.28	0.134
	Genetic	1.02	0.90–1.17	0.27
	Smoking DNAm	1.46	1.29–1.65	1.4 × 10^−9^
Education	Phenotypic	0.93	0.81–1.08	0.352
	Epigenetic	1.00	0.85–1.17	0.979
	Genetic	0.95	0.82–1.10	0.474
	Smoking DNAm	1.48	1.29–1.70	1.8 × 10^−8^
BMI	Phenotypic	1.23	1.06–1.42	0.005
	Epigenetic	1.04	0.90–1.20	0.614
	Genetic	1.09	0.94–1.26	0.263
	Smoking DNAm	1.57	1.39–1.78	8.3 × 10^−13^
Total cholesterol	Phenotypic	0.85	0.73–0.99	0.031
	Epigenetic	0.95	0.81–1.12	0.542
	Genetic	1.11	0.96–1.27	0.155
	Smoking DNAm	1.49	1.32–1.68	9.7 × 10^−11^
HDL cholesterol	Phenotypic	0.92	0.77–1.09	0.318
	Epigenetic	0.92	0.78–1.08	0.307
	Genetic	1.08	0.94–1.25	0.277
	Smoking DNAm	1.48	1.31–1.67	3.0 × 10^−10^
LDL with remnant cholesterol	Phenotypic	0.89	0.77–1.03	0.122
	Epigenetic	0.99	0.84–1.17	0.910
	Genetic	1.08	0.93–1.25	0.303
	Smoking DNAm	1.49	1.31–1.68	3.3 × 10^−10^
Total:HDL cholesterol ratio	Phenotypic	0.99	0.85–1.15	0.870
	Epigenetic	1.12	0.95–1.30	0.170
	Smoking DNAm	1.48	1.30–1.67	6.9 × 10^−10^
Waist-to-hip ratio	Epigenetic	1.20	1.04–1.39	0.012
	Genetic	0.94	0.82–1.07	0.361
	Smoking DNAm	1.47	1.31–1.66	1.3 × 10^−10^
Body fat percentage	Epigenetic	1.11	0.96–1.27	0.147
	Genetic	1.19	1.04–1.36	0.013
	Smoking DNAm	1.51	1.34–1.70	1.1 × 10^−11^

Cox proportional hazards outputs are presented for models adjusting for age, sex, phenotypes (where applicable), polygenic scores (where applicable), white blood cell counts, and smoking DNAm scores. All effect sizes are per standard deviation

## Discussion

We have identified DNA methylation-based predictors for ten modifiable lifestyle and health factors that: (1) explain varying degrees of proportions of their phenotypic variance and do so independently from corresponding genetic predictors; (2) help to characterize individual differences; and (3) show association with a clinically relevant outcome through prediction of mortality and do so independently from phenotypic and genetic measures.

The DNAm predictors explained different proportions of the variance in the modifiable complex traits, from 0.6% for LDL with remnant cholesterol up to 60.9% for smoking. By combining genetic and epigenetic predictors we were able to augment these predictions. The previous best estimate for genetic plus epigenetic BMI prediction was ~ 15% [14]. The combined predictor in the current study was able to explain nearly 20% of the variance in BMI. The alcohol consumption, HDL cholesterol, and smoking predictions were largely driven by the DNAm predictors whereas the LDL with remnant cholesterol prediction was largely driven by the genetic predictor for LDL cholesterol.

There is near-perfect discrimination between current and never smokers based on the smoking DNAm predictor and moderate discrimination between obese individuals, moderate-to-heavy drinkers, and individuals with high HDL cholesterol levels. Differentiating those with a high level of education is more a function of genetics than DNAm, although the combined predictive power remains poor. In the case of some phenotypes, the varying discriminatory abilities of their DNAm scores may be attributed to degree, duration, and/or time of exposure. Misclassification of lighter drinkers as heavy drinkers based on DNAm score may be reflective of effects of recent or infrequent above-average alcohol consumption. This highlights a potential application of DNAm-based signatures as proxies for self-reported phenotypes. In the case of current smokers, cigarette smoke is likely to be a constant exposure up to the time of sampling, which may reflect the high sensitivity of the DNAm-based smoking score. Former smokers display a DNAm score intermediate score relative to that of current and never smokers, which may reflect a degree of temporality in the smoking DNAm score (Additional file 2: Figure S7). Application of these predictors alongside existing DNAm-based age predictors [17, 18] may also be of use in forensic investigations, given an unknown blood sample [19].

As with the previous EWAS analysis of education [5], there is a strong overlap with smoking-related methylation signals. The strength of the correlation between the education and smoking DNAm predictors (*r* = − 0.49) is particularly interesting when placed in context with their more modest phenotypic correlation (*r* = − 0.14). Given that DNA methylation is highly predictive of smoking status [9], it may be the case that, should a single smoking-sensitive CpG feature in a DNAm predictor for another trait—here, education—then this drives a high correlation between the two DNAm predictors. Notably, previously reported DNAm-based biomarkers of BMI (e.g. cg11024682 [7]), total cholesterol (e.g. cg16000331 [11]), smoking (e.g. cg05575921 [9]), and HDL cholesterol (e.g. cg17901584 [10]) were among the features with the largest absolute coefficients in their respective models. It is also of note that the DNAm predictor for education contained established DNAm-based biomarkers of smoking from the *AHRR* gene (cg11902777 and cg05575921 [9]). A DNAm education predictor excluding this feature/CpG was strongly correlated with the primary predictor (*r* = 0.996). Correlations between different CpG features within each of the DNAm predictors may be responsible for the association observed between predictors.

The survival analysis in the out-of-sample prediction LBC1936 cohort yielded significant associations for the smoking, alcohol, waist-to-hip ratio, and education DNAm predictors. When included as a covariate, the smoking DNAm predictor attenuated the DNAm-mortality associations for both the education and alcohol predictors, but not the predictor for waist-to-hip ratio. In the case of phenotypic alcohol consumption and education, there were no associations with all-cause mortality. This may suggest these scores are capturing additional factors related to their corresponding phenotypes (such as smoking), which may have more direct biological consequences that contribute to risk of mortality. The DNAm score for education, for example, was correlated with phenotypic smoking status. Consistent with our phenotype-based survival analyses, others have reported positive associations between mortality risk and smoking [20, 21] whereas higher educational attainment and old-age total cholesterol levels have been associated with a decreased mortality risk [22–25]. Moreover, a recent meta-analysis failed to find a significant relationship between phenotypic alcohol consumption and all-cause mortality [26].

It should be noted that the polygenic score used to predict LDL with remnant cholesterol was derived from a GWAS of LDL cholesterol only. Both DNAm and genetic scores explained a small proportion of the variance in LDL with remnant cholesterol while the predictive performance of high versus low LDL with remnant cholesterol (based on guidelines for LDL cholesterol only) was poor. It is possible that the heterogeneity of the phenotype (calculated from the difference between measured total and HDL cholesterol) posed a limitation in the development of a reliable DNAm-based signature. Developing DNAm-based predictors of LDL and remnant cholesterol using separate measurements of LDL and remnant cholesterol may be a more successful strategy for future studies.

There are two key strengths to this study. First, the sample size of the GS cohort, which is one of the single largest epidemiological cohort studies with DNA methylation data, enabled us to improve on previous DNAm predictors by: modelling all CpG sites simultaneously; training the predictor using cross-validation penalized regression modelling; and reducing heterogeneity in both phenotypic and methylation measurement through a single data collection and analysis protocol. Second, we could predict not only the relevant phenotypes of interest but also a clinically meaningful outcome (mortality) in our large, genetically homogenous, out-of-sample prediction cohort, LBC1936. Other studies with DNA methylation data and longitudinal disease follow-up for, for example, cardiometabolic, cardiovascular, and cancer-related outcomes will be able to further test the predictive performance of our DNAm predictors.

The GS cohort contained related individuals who may be more phenotypically similar for the traits under investigation. Residuals from sensitivity analyses that adjusted the phenotypes for pedigree structure as a random effect, in addition to age, sex, and population stratification as fixed effects, correlated highly (minimum Pearson *r* = 0.96) to those from the models without pedigree adjustment. The older age range of LBC1936 and longitudinal follow-up enabled us to examine the ability of DNAm-based predictors for complex traits to predict mortality, independently of the phenotypes themselves. As mentioned previously, the test cohort was older, had fewer years of education, were lighter drinkers, heavier smokers relative to the training cohort, had lower levels of total cholesterol and LDL (with remnant) cholesterol, and a lower total:HDL cholesterol ratio. The DNAm predictors may perform differently on these measures in cohorts that are more analogous in age and phenotypic distribution to the training dataset, GS.

## Conclusions

In summary, we showed that DNAm predictors are able to predict modifiable health and lifestyle factors with some success. They can also augment phenotypic prediction of mortality. Future studies should focus on other incident health outcomes, such as cardiometabolic disease and cancer. There is scope to use these DNAm predictors, in addition to DNAm-based predictors of age, to help identify lifestyle characteristics from DNA.

## Methods

### Training dataset for the DNAm predictors: Generation Scotland

The DNAm predictors were built on a subset of 5087 individuals from GS, who had DNA methylation measured as part of a sub-study: Stratifying Resilience and Depression Longitudinally (STRADL). The parent cohort, GS, contains detailed cognitive, physical, health, and genetic data on over 22,000 individuals from across Scotland, aged 18–99 years [27, 28]. It is a family-structured, population-based longitudinal cohort study. Stored DNA samples from bloods collected at the study baseline (2006–2011) were used for the DNAm analysis.

### Methylation preparation in Generation Scotland

Quality control was performed on Illumina HumanMethylationEPIC BeadChip DNA methylation data from blood samples of 5200 individuals from the GS cohort. Details have been reported previously [29]. Three individuals who had answered “yes” to all self-reported conditions were excluded from the analysis. Filtering for outliers, sex mismatches, non-blood samples, poorly detected probes, and samples was performed. A full description is provided in Additional file 4. Further filtering was then carried out to remove CpGs with missing values, non-autosomal and non-CpG sites, and any sites not present on the Illumina 450 k array. The latter criterion enabled prediction into the LBC study.

### Phenotype preparation in Generation Scotland

We considered ten phenotypes from GS for the analysis: educational attainment; BMI; total cholesterol; HDL cholesterol; LDL with remnant cholesterol; total:HDL cholesterol ratio; waist-to-hip ratio; percentage body fat; and self-reported alcohol consumption and smoking status. Phenotypes for LDL with remnant cholesterol were calculated as the difference between total cholesterol and HDL cholesterol. Educational attainment was assessed on an ordinal scale, the other traits were assessed as continuous traits and in their standard units of measurement with pack years for smoking and units per week for alcohol (full details in Additional file 4).

Each phenotype was then regressed on age, sex, and ten genetic principal components [30] with the residuals being entered as the dependent variable in the LASSO models.

### LASSO regression in Generation Scotland

Penalized regression models were run using the glmnet library in R [31, 32]. Tenfold cross-validation was applied and the mixing parameter (alpha) was set to 1 to apply a LASSO penalty. Coefficients for the model with the lambda value corresponding to the minimum mean cross-validated error were extracted and applied to the corresponding CpGs in an out of sample prediction cohort to create the DNAm predictors.

### The out-of-sample prediction cohort: Lothian Birth Cohort 1936

LBC1936 [33, 34] was used for external DNAm predictions. LBC1936 is a cohort comprising individuals born in 1936, who were aged approximately 70 years at recruitment. Here, DNAm was assessed in blood samples from wave 1 of the study between 2004 and 2007.

### Methylation preparation in the Lothian Birth Cohort 1936

DNAm from whole blood was assessed in the LBC1936 using the Illumina 450 k methylation array. Over 90% of the 450 k CpG sites are present on the EPIC array. Quality control details have been reported previously [35] and are detailed in Additional file 4.

### Polygenic scoring in the Lothian Birth Cohort 1936

Polygenic scores were created in LBC1936 using PRSice [36] with clumping parameters of R^2^ > 0.25 over 250-kb sliding windows. Genotyped data were generated at the Wellcome Trust Clinical Research Facility using the Illumina 610-Quadc1 array (San Diego, CA, USA). The SNP weights for all variants (*p* < 1) for the traits [37–43] were taken from large genome-wide association studies (GWAS). Where LBC1936 was included in the discovery GWAS (educational attainment [40]), the meta-analysis was re-run after its exclusion.

### Phenotypes in the Lothian Birth Cohort 1936

Phenotype measurement details in LBC1936 are as follows: self-reported smoking status (current smoker, ex-smoker, never smoked); alcohol consumption in a typical week (recoded into units); and education (years of full-time education) were assessed along with BMI (defined as the ratio of weight in kg divided by height in m^2^); total cholesterol; HDL cholesterol; LDL with remnant cholesterol (all in mmol/L); total:HDL cholesterol ratio; waist-to-hip ratio; and percentage body fat. LDL with remnant cholesterol was defined as the difference between total cholesterol and HDL cholesterol. Binary categorizations of smoking (current versus never), BMI (> 30 vs ≤ 30 kg/m^2^, defined as obese and non-obese, respectively), education (> 11 vs ≤11 years, which is roughly equivalent to a college education level for LBC1936), and alcohol consumption were used as outcomes for receiver operating characteristic (ROC) curve analyses. Cholesterol-related variables were dichotomized as high or low based on NHS guidelines on cholesterol levels (https://www.nhs.uk/conditions/high-cholesterol/): > 5 mmol/L for total cholesterol, > 3 mmol/L for LDL with remnant cholesterol, > 1 mmol/L for HDL cholesterol, and ≥ 4 for total:HDL cholesterol ratios. Sex-specific dichotomizations were applied to the alcohol consumption phenotype, as per UK health recommendations at the time of data collection (≤ 21 vs > 21 units per week for men, and ≤ 14 vs > 14 units per week for women; corresponding to moderate and heavy alcohol consumption in each gender, respectively. Mortality data were obtained through data linkage to the National Health Service Central Register, provided by the General Register Office for Scotland (now National Records of Scotland). The mortality data used in the present analysis were correct as of January 2018.

### Prediction analysis in the Lothian Birth Cohort 1936

Area under the curve (AUC) estimates were estimated for binary categorizations of BMI, smoking, alcohol consumption, college education, and cholesterol variables. Linear regression models were used to identify the proportion of phenotypic variance explained by the corresponding DNAm predictor and to determine whether this was independent of the polygenic (genetic) signal for each phenotype. Ordinal logistic regression was used for the categorical smoking variable (never, ex, current smoker). Age and sex were considered as covariates, the phenotypic measure was the dependent variable, and the polygenic score or DNAm predictor were the independent variables of interest. Incremental R^2^ estimates were calculated between the null model and the models with the predictors of interest. An additive genetic and epigenetic model for BMI in the LBC1936 has been reported previously, although a different DNAm predictor, based on unrelated individuals, was derived from the GS data [44]. ROC curves were developed for smoking status, obesity, high/low alcohol consumption, college education and cholesterol variables, and AUC estimates were estimated for binary categorizations of these variables using the pROC library in R [45]. Cox proportional hazards survival models [46] were used to examine whether the phenotype, polygenic score, or DNAm predictor explained mortality risk and if they do so independently of one another. Sex was included as a covariate in all models. Correction for multiple testing was applied using the Bonferroni method.

## Additional files

Additional file 1: DNAm signature CpGs and corresponding weights for BMI (**Table S1**), smoking (**Table S2**), alcohol consumption (**Table S3**), educational attainment (**Table S4**), total cholesterol (**Table S5**), HDL cholesterol (**Table S6**), LDL (with remnant) cholesterol (**Table S7**), Total:HDL cholesterol ratio (**Table S8**), waist-to-hip ratio (**Table S9**), and percentage body fat (**Table S10**). (XLSX 159 kb)

Additional file 2: **Figure S1.** Correlations between phenotypes in GS samples. **Figure S2.** Correlations between phenotypes in LBC1936 samples. **Figure S3.** Correlations between DNA methylation scores in LBC1936 samples. **Figure S4.** Correlations between genetic scores in LBC1936 samples. **Figure S5.** HRs for phenotypic predictors of mortality in LBC1936 samples. **Figure S6.** HRs for polygenic predictors of mortality in LBC1936 samples. **Figure S7.** DNA methylation scores for current, former, and never smokers in LBC1936. (PDF 382 kb)

Additional file 3: **Table S11.** Prediction of traits with and without genetic scores. **Table S12.** Cox proportional hazards survival models output for phenotypic, epigenetic (DNAm), and genetic (polygenic) predictors of health and lifestyle factors. (XLSX 14 kb)

Additional file 4: Document contains further information on phenotype preparation and quality control of DNAm data for GS and LBC1936. (PDF 90 kb)

## Abbreviations

AUC: Area under the curve
BMI: Body mass index
CI: Confidence interval
CpG: Cytosine-phosphate-guanine dinucleotide
DNAm: DNA methylation
EWAS: Epigenome-wide association study
GS: Generation Scotland: The Scottish family health study
HDL: High-density lipoprotein
HR: Hazard ratio
LASSO: Least absolute shrinkage and selector operator
LBC1936: Lothian Birth Cohort 1936
LDL: Low-density lipoprotein
STRADL: Stratifying resilience and depression longitudinally
WHR: Waist-to-hip ratio
