# Supplementary material for: Epigenetic prediction of complex traits and death
Source: Genome Biol. 2018 Sep 27;19:136. doi: 10.1186/s13059-018-1514-1 (PMC6158884; doi:10.1186/s13059-018-1514-1)

Figure S1: Correlations between phenotypes in Generation Scotland samples

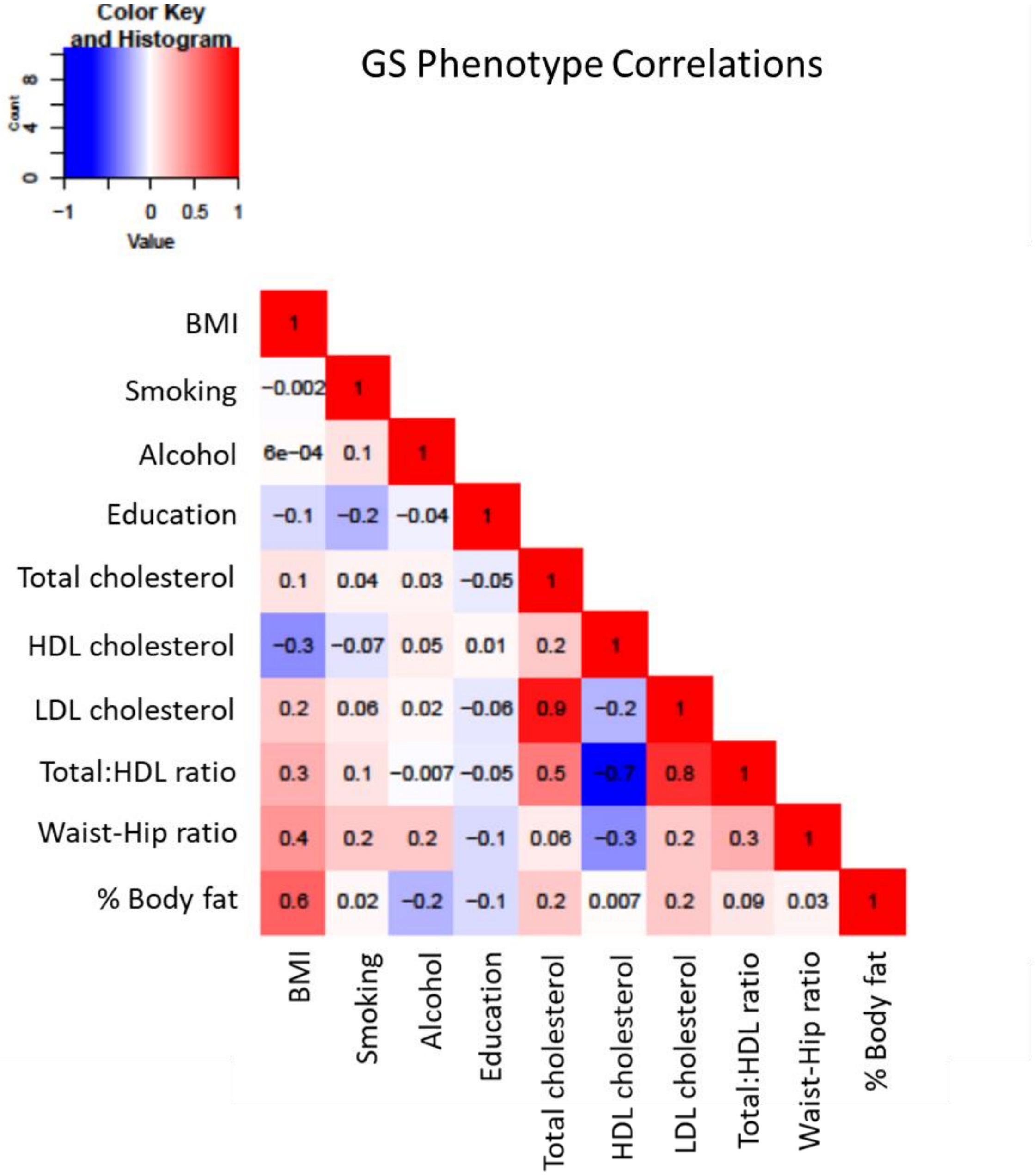

Figure S2: Correlations between phenotypes in LBC1936 samples

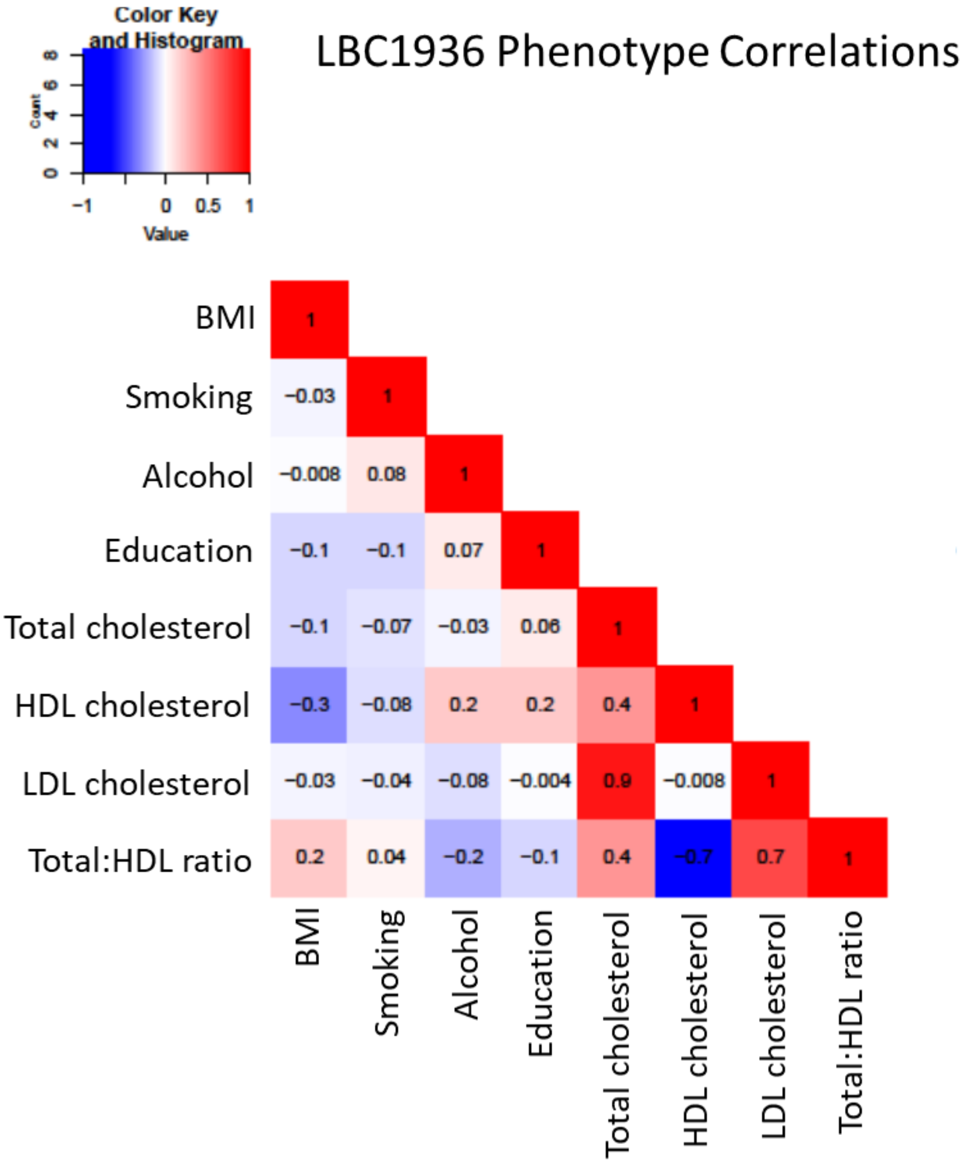

Figure S3: Correlations between DNA methylation scores in LBC1936 samples

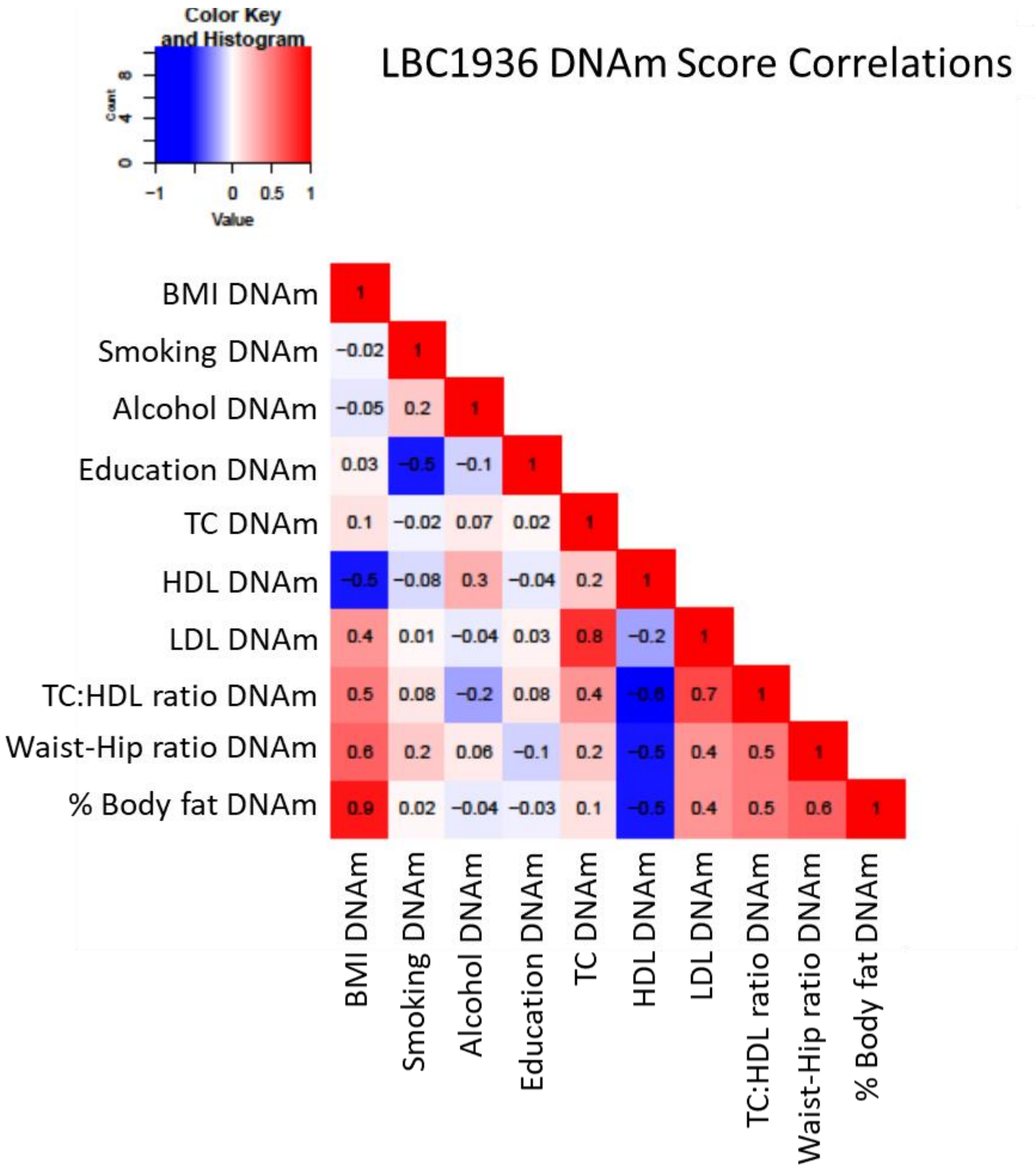

Figure S4: Correlations between genetic scores in LBC1936 samples

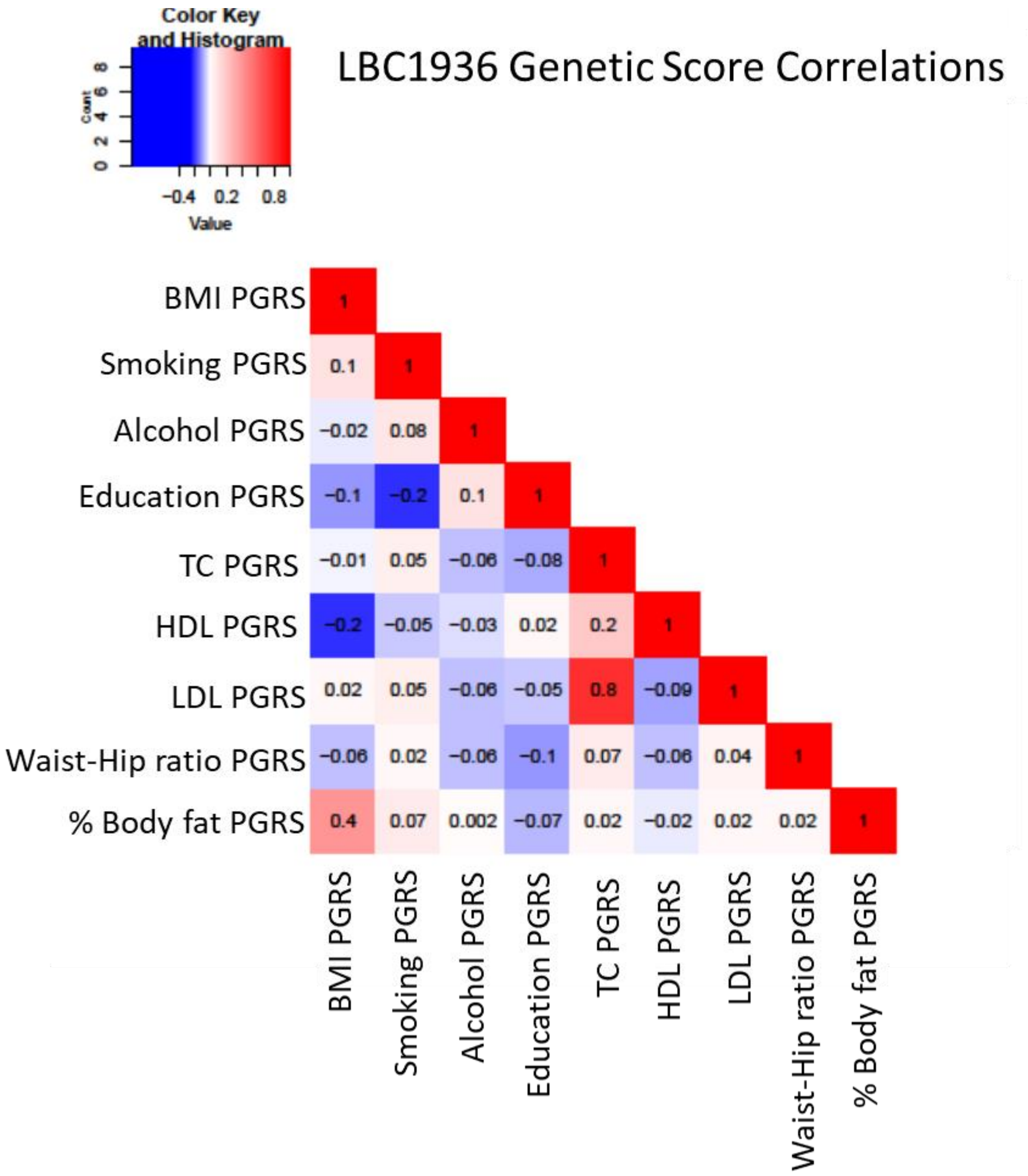

Figure S5: Hazard ratios for phenotypic predictors of mortality in LBC1936 samples

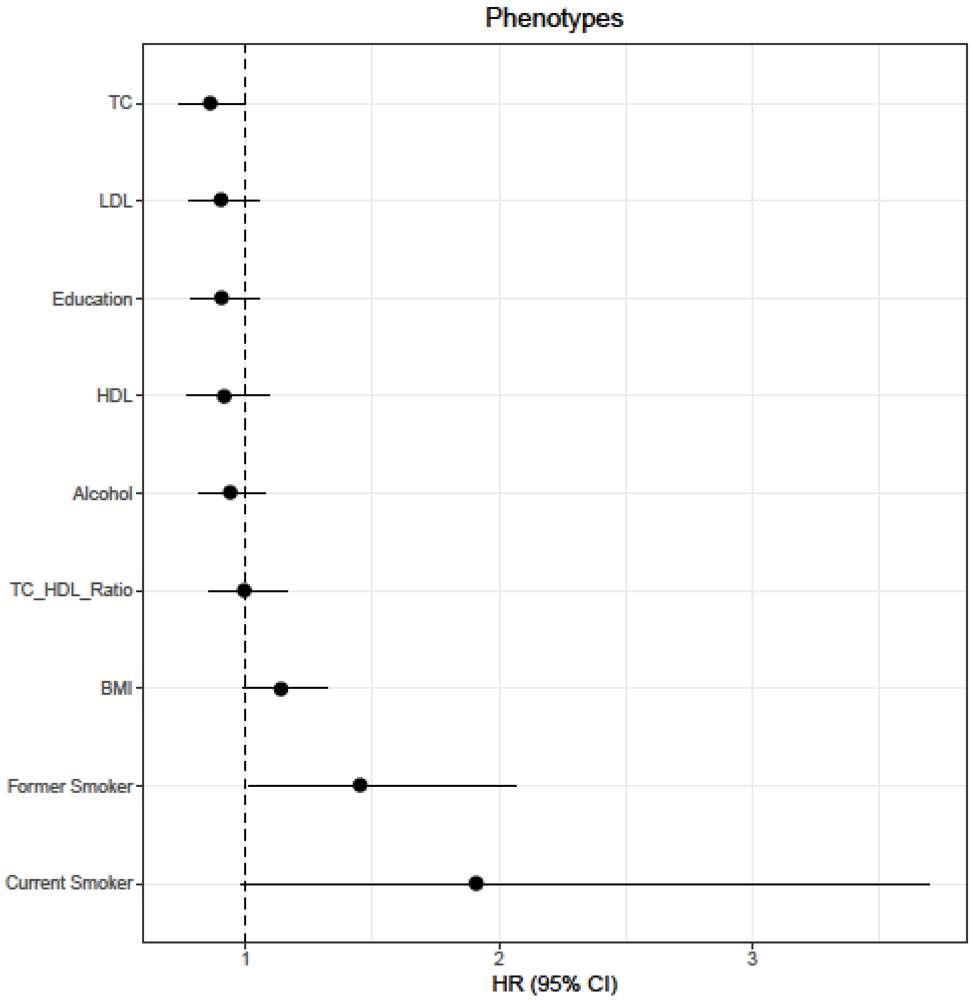

Figure S6: Hazard ratios for polygenic predictors of mortality in LBC1936 samples

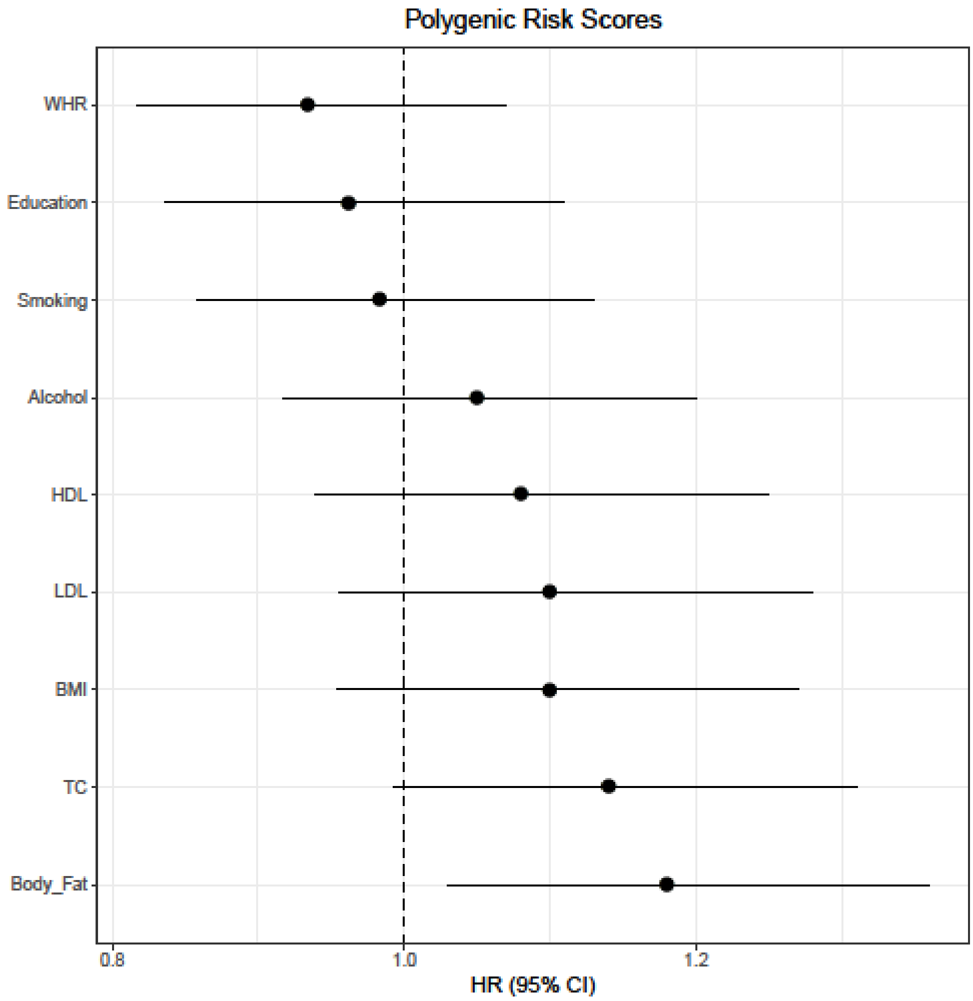

Figure S7: DNAm scores for current, former and never smokers in LBC1936

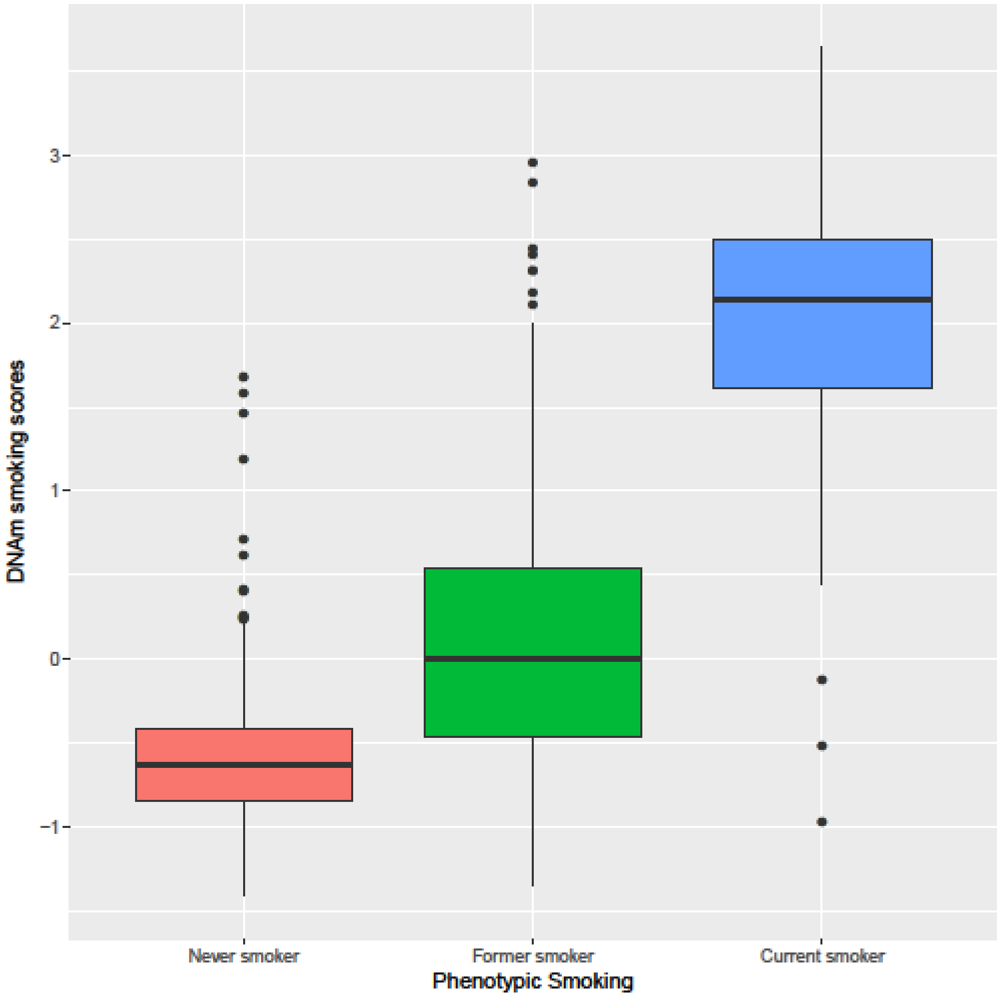

Supplement: Supplementary file 2 — Figure S1. Correlations between phenotypes in GS samples. Figure S2. Correlations between phenotypes in LBC1936 samples. Figure S3. Correlations between DNA methylation scores in LBC1936 samples. Figure S4. Correlations between genetic scores in LBC1936 samples. Figure S5. HRs for phenotypic predictors of mortality in LBC1936 samples. Figure S6. HRs for polygenic predictors of mortality in LBC1936 samples. Figure S7. DNA methylation scores for current, former, and never smokers in LBC1936. (PDF 382 kb) [file 13059_2018_1514_MOESM2_ESM.pdf]
