# Supplementary material for: Epigenetic prediction of complex traits and death
Source: Genome Biol. 2018 Sep 27;19:136. doi: 10.1186/s13059-018-1514-1 (PMC6158884; doi:10.1186/s13059-018-1514-1)
Supplement: Supplementary file 4 — Document contains further information on phenotype preparation and quality control of DNAm data for GS and LBC1936. (PDF 90 kb) [file 13059_2018_1514_MOESM4_ESM.pdf]

Quality control of Generation Scotland DNA methylation data

Visual inspection of a plot of log median intensity of methylated versus unmethylated signal [1] was used to identify outliers, which were excluded. Sample exclusions were also made where predicted sex, based on DNA methylation data, did not match the sex recorded in the GS database. Finally, samples were excluded if ≥1% of CpGs had a detection p-value in excess of 0.05. Probes were excluded if they had a beadcount below 3 in at least 6 samples and when ≥0.5% of samples had a detection p-value >0.05. This left data available for 860,926 methylation sites in 5,087 participants.

Further filtering was performed to remove (1) any sites with missing values, (2) non-autosomal sites, (3) non-CpG sites, and (4) CpG sites not present on the Illumina 450k array. Criterion (4) enables prediction into existing datasets as the majority of the CpG sites on the 450k array are present on the EPIC array.

Quality control of DNA methylation data in the Lothian Birth Cohort 1936

After background correction, probes were removed if they were poorly detected (P>0.01) in >5% of samples or of low quality (via manual inspection). Samples were removed if they had a low call rate (P<0.01 for <95% of probes), a poor match between genotype and SNP control probes, or incorrect DNAm-predicted sex.

Phenotype preparation in Generation Scotland

Educational attainment was measured via an ordinal scale: 0: 0 years, 1: 1-4 years, 2: 5-9 years, 3: 10-11 years, 4: 12-13 years, 5: 14-15 years, 6: 16-17 years, 7: 18-19 years, 8: 20-21 years, 9: 22-23 years, 10: ≥24 years of full-time education. It was treated as a continuous

variable for the current analyses. BMI, assessed as the ratio of weight in kilograms to height in metres squared (kg/m^2), was trimmed for extreme values (<17 and >50 kg/m^2) before being log transformed. Alcohol was assessed in units per week and was only considered in those who reported that their intake as representative of a normal week. To reduce skewness in the distribution of alcohol consumption, a log(units + 1) transformation was performed. The addition of the constant retains non-drinkers, who reported a consumption of 0 units. Smoking was assessed in pack years (calculated by multiplying the number of packs smoked per day by the number of years the participant has smoked) for current and never smokers; ex-smokers were excluded due to complications in adjusting for time since cessation into the pack years calculation. Those who reported starting at age 10 or under were treated as data entry or self-report errors and were excluded along with non-smokers who had a non-zero (impossible) entry for pack years. As with the alcohol phenotype, a log(pack years + 1) transformation was used to reduce skew, leaving between 2,819 and 5,036 individuals, depending on phenotype (**Table 1**).

1. Fortin J-P, Fertig E, Hansen K. shinyMethyl: interactive quality control of Illumina 450k DNA methylation arrays in R. F1000Research. 2014. doi:10.12688/f1000research.4680.2.
